# Perceptual learning deficits mediated by somatostatin releasing inhibitory interneurons of olfactory bulb in an early life stress mouse model

**DOI:** 10.1038/s41380-023-02244-3

**Published:** 2023-09-19

**Authors:** Meenakshi Pardasani, Anantha Maharasi Ramakrishnan, Sarang Mahajan, Meher Kantroo, Eleanor McGowan, Susobhan Das, Priyadharshini Srikanth, Sanyukta Pandey, Nixon M. Abraham

**Affiliations:** Laboratory of Neural Circuits and Behaviour (LNCB), Department of Biology, Indian Institute of Science Education and Research (IISER), Pune, Maharashtra 411008 India

**Keywords:** Neuroscience, Depression, Biological techniques

## Abstract

Early life adversity (ELA) causes aberrant functioning of neural circuits affecting the health of an individual. While ELA-induced behavioural disorders resulting from sensory and cognitive disabilities can be assessed clinically, the neural mechanisms need to be probed using animal models by employing multi-pronged experimental approaches. As ELA can alter sensory perception, we investigated the effect of early weaning on murine olfaction. By implementing go/no-go odour discrimination paradigm, we observed olfactory learning and memory impairments in early life stressed (ELS) male mice. As olfactory bulb (OB) circuitry plays a critical role in odour learning, we studied the plausible changes in the OB of ELS mice. Lowered c-Fos activity in the external plexiform layer and a reduction in the number of dendritic processes of somatostatin-releasing, GABAergic interneurons (SOM-INs) in the ELS mice led us to hypothesise the underlying circuit. We recorded reduced synaptic inhibitory feedback on mitral/tufted (M/T) cells, in the OB slices from ELS mice, explaining the learning deficiency caused by compromised refinement of OB output. The reduction in synaptic inhibition was nullified by the photo-activation of ChR2-expressing SOM-INs in ELS mice. The role of SOM-INs was revealed by learning-dependent refinement of Ca^2+^dynamics quantified by GCaMP6f signals, which was absent in ELS mice. Further, the causal role of SOM-INs involving circuitry was investigated by optogenetic modulation during the odour discrimination learning. Photo-activating these neurons rescued the ELA-induced learning deficits. Conversely, photo-inhibition caused learning deficiency in control animals, while it completely abolished the learning in ELS mice, confirming the adverse effects mediated by SOM-INs. Our results thus establish the role of specific inhibitory circuit in pre-cortical sensory area in orchestrating ELA-dependent changes.

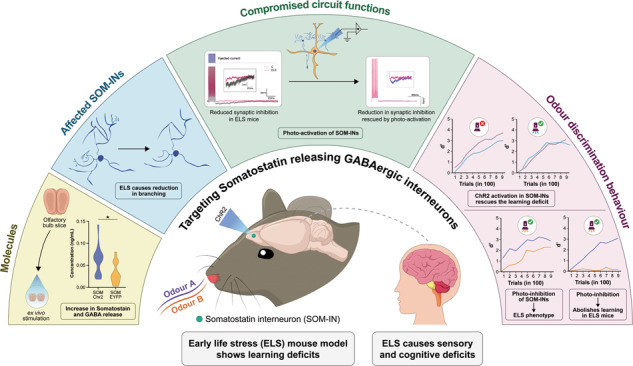

## Introduction

Early life adversity (ELA) brings about neural circuit alterations, which can elicit dysfunctional physiological and behavioural outcomes [1, 2]. Such negative effects pose challenges on the emotional, sensory and cognitive capacities of an individual [1–5]. Humans with history of stressful experiences during childhood and major depressive disorders (MDD), suffer from sensory and cognitive deficits [6]. To unravel the neural underpinnings and to dissect out the causality between circuits and disorder phenotypes, brain areas that are capable of encoding sensation as well as cognition can be directly studied in animal models. Probing the effect of early life stress (ELS) on specific regions that can control perceptual decision-making will facilitate gaining mechanistic insights and thereby enable the development of therapeutic interventions.

Somatosensory perception is shown to be negatively affected in mouse model with postnatal stress [7, 8]. Although it is known that olfactory centres are entwined with limbic brain regions, olfactory dysfunctions under conditions of ELA remain elusive. Mammalian olfactory bulb (OB) is a specialised pre-cortical area of the brain that processes smell and can be modulated by the affective states. Removal of OB, which leads to depressive phenotypes, can result in malfunctions in downstream cortical-hippocampal-amygdala network in rats [9–11]. OB volume, a correlate for olfactory functionality, has been found to be reduced in patients with MDD [12]. In adult humans with history of childhood maltreatment, which is a type of ELA, OB volume was reduced by 20% with a concomitant decrease in their olfactory detection thresholds and problems in identifying odours [13]. In mouse model of anxiety/depression-inducing states generated by chronic corticosterone treatment, impairments in olfactory learning were observed [14]. Even stressed-reared juvenile rats displayed altered responses in odour aversive learning [15]. This suggests that different stressors or adversities can modify bulbar activity, which may result in exacerbated behavioural responses.

Our interest lied in investigating how a deleterious change in the early rearing environment, by weaning the mice as early as postnatal day 14 (P14) can have consequences on olfactory perception and decision-making in adult life. Early postnatal life is a crucial time as OB neurons undergo morphological, electrophysiological and functional maturation [16]. OB displays high levels of plasticity in response to experiences such as enrichment, reinforcement learning and stress by regulating inhibition on the projection neurons, the Mitral/Tufted (M/T) cells. [17–19]. One of the interneuron types in the OB, somatostatin (SOM)-releasing, Gamma-aminobutyric acid (GABA)-ergic ones (SOM-INs), has been demonstrated in providing neuromodulatory effect via the somatostatin receptors (SSTRs)- 1 to 4 [20]. SOM is involved in cognition and reduced in several neuropsychiatric disorders across patients and animal models [21, 22]. We decided to use SOM-transgenic mice to probe the effect of ELA on this micro-circuitry and thereby its role in olfactory perception. SOM-INs are present across the inner part of external plexiform layer (EPL), sparsely in glomerular layer (GL) as well as in deep layers of granule cell layer (GCL) [23]. They may participate in both narrow and broad range reciprocal inhibitory processes, possibly function to provide gain control to M/T cells via action of both GABA and SOM starting from the early stages of olfactory processing, and modify during learning and memory [20, 24, 25]. Using retrograde labelling, bulbar SOM-INs have been recently shown to receive centrifugal projections from lateral amygdala, hippocampus, piriform and entorhinal cortices, regions that are capable of being modified under conditions of adversity during early life [20]. Such characteristics make these neurons an interesting candidate to investigate the effects induced by ELS.

In this work, we explore the behavioural, neuro-anatomical, electrophysiological and functional correlates of ELA induced changes in the OB-controlled perceptual learning deficits by using the mouse model of early weaning. By utilising optogenetics, we dissect out the causal role of SOM-INs in controlling animals’ learning efficiency. Further, we demonstrate the rescue of learning deficits observed in ELS mice by photoactivating these interneurons.

## Materials and methods

### Subjects

A total of 40 normally weaned (control, C, males) and 32 ELS, C57BL/6J (The Jackson’s laboratory, males) were utilised for the behavioural experiments. Set 1 consisting of 21 control and 17 ELS mice were utilised for carrying out go/no-go odour discrimination task for a series of 7 odour pairs and 2 memory tasks and open field test during late adulthood. A subset of Set 1 mice was further utilised for carrying out odour discrimination task during late adulthood. Set 2 mice consisting of 11 control and 8 ELS mice were utilised for carrying out the open field and buried food pellet (BFP) test and further for carrying out odour discrimination at different dilutions, all done during young adulthood (carried out between PND60 and PND100). Set 3 comprising of 8 control and 7 ELS mice were utilised for novel object recognition task and were subsequently trained for carrying out the complex carvones enantiomers discrimination task. 3 trained mice belonging to each group from set 3 were used for c-Fos quantification experiment.

SOM-transgenic mice were obtained by crossing the following genotypes that were obtained from The Jackson’s laboratory [26];

- SOM-EYFP : B6.Cg-Sst^tm2.1(cre)Zjh^/J (SST-IRES-Cre, 013044) with B6.CgGt(ROSA)26Sor^tm1(EYFP)Cos^/J (R26R-EYFP, 006148)
- SOM-ChR2(-EYFP) : B6.Cg-Sst^tm2.1(cre)Zjh^/J (SST-IRES-Cre, 013044) with B6.CgGt(ROSA)26Sor^tm32(CAGCOP4*H134R/EYFP)Hze^/J (Ai32, 024109)
- SOM-Arch(-GFP) : B6.Cg-Sst^tm2.1(cre)Zjh^/J (SST-IRES-Cre, 013044) with B6.CgGt(ROSA)26Sor^tm35.1(CAG-aop3/GFP)Hze^/J (Ai35D, 012735).

A total of 44 SOM-transgenic control and 49 ELS mice (SOM-EYFP, SOM-ChR2 and SOM-Arch, males) were utilised for anatomical, behavioural, electrophysiological and optogenetic experiments. Set 4 consisting of 3 Control and 3 ELS mice were used for morphological analyses of the SOM-INs. Set 5 comprising of 8 control and 7 ELS mice were utilised for obtaining current clamp recordings from the OB. Additionally, 7 SST-ChR2 ELS mice were utilised for opto-physiological recordings in presence and absence of photo-stimulation. 11 SOM-EYFP (C + ELS) and 11 SOM-ChR2 (C + ELS) mice belonging to Set 6 were used for ex vivo photo-stimulation experiments pertaining to quantification of SOM neuropeptide and GABA neurotransmitter. For optogenetic modifications during behavioural training experiments; Set 7 comprising of 8 SOM-EYFP, 7 SOM-ChR2 and 7 SOM-Arch control mice as well as 8 SOM-EYFP, 7 SOM-ChR2 and 6 SOM-Arch ELS mice were utilised. For Miniscope based calcium imaging from SOM-interneurons, 6 SOM-GCaMP control and 6 SOM-GCaMP ELS mice belonging to Set 8, were utilised. Additionally, 1 SOM-GCaMP mouse was utilised for confocal imaging of these neurons in different layers of the OB. These mice were obtained by crossing _B6.Cg-Sst_^tm2.1(cre)Zjh^/J *(SST-IRES-Cre, 013044)* with B6;129S-Gt(ROSA)26Sor^tm95.1(CAG-GCaMP6f)Hze^/J *(Ai95D, 024105)* to obtain the desired phenotype.

Inclusion/Exclusion Criteria: Only healthy, optimally motivated animals were used for all experiments. If we observed a clear over/under motivation reflected in the licking behaviour of mice during the behaviour experiments [27, 28] or technical failures in optogenetic experiments, mice were excluded in certain data sets, which was never more than 10−15% of reported final numbers. No specific randomisation was used in different experimental groups. However, animals were counterbalanced towards rewarded/non-rewarded stimuli used in behavioural experiments.

Twelve-hour light/dark cycle was maintained and mice were grouped in individually ventilated cages in a temperature- and humidity-controlled animal facility. Animal care as well as the procedures were in agreement with Institutional Animal Ethics Committee (IAEC) at IISER Pune and the Committee for the Control and Supervision of Experiments on Animals (CCSEA), Government of India.

### Early weaning

**Fig. 1:**
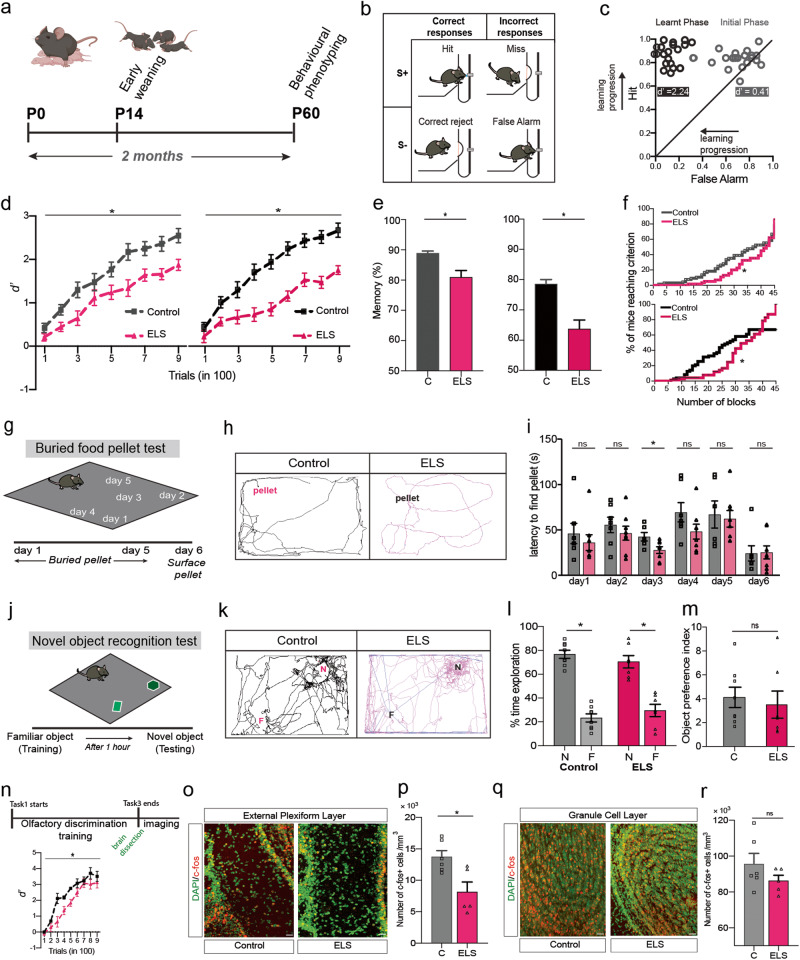
Olfactory specific perceptual learning and memory deficits in ELS mice. **a** Early weaning and experimental timeline. **b** Responses of the mouse in a Go/No-go olfactory discrimination paradigm. **c** Each point indicates a mouse shown during their initial phase (grey circles) and final phase of learning (black circles) indicating a shift towards accurate performance (d-prime (d’) value increased) as the training proceeded. **d** d’ value is significantly lower on a simple odour pair discrimination task (AP vs. ON) for ELS mice as compared to control mice (*n*_C_ = 21, *n*_ELS_ = 17; *p* = 0.0013, *F* = 12.08, Two-way ANOVA, LSD Fisher test). Impaired olfactory learning in ELS mice was also observed in a complex binary mixture task of AP vs. ON, shown by significnatly lower d’ over training (*n*_C_ = 21, *n*_ELS_ = 16; *p* < 0.0001, *F* = 30.2, Two-way ANOVA, LSD Fisher test). **e** Significant reduction in olfactory memory for AA vs. EB (*n* = 9 for both groups, *p* = 0.0037, Unpaired t-test, two-tailed) and its binary mixture pair (*n*_c_ = 14, *n*_ELS_ = 13, *p* = 0.023, Unpaired *t* test, two-tailed) in ELS mice. **f** The percentage of mice belonging to ELS group that reached the criterion performance (80% or above and not dropping below 65% thereafter) took significantly higher number of training blocks (20 trials per block) as compared to control mice, over the first two simple odour pairs carried out in the sequence of training (AP vs. ON and MB vs. NN) (χ^2^ = 4.14, *p* = 0.04, Gehan−Breslow−Wilcoxon test). Over the first two complex odour pairs (AP vs. ON 60-40 and AA vs. EB 60-40), the criterion performance reached was far less for ELS mice (χ^2^ = 9.04, *p* = 0.002, Gehan-Breslow-Wilcoxon test). **g** Buried food pellet test was carried out for 5 days. On day 6, surface pellet test was done as a control test. Diagram shows the position of the buried pellet across five days. **h** Tracks taken by control and ELS mice to search for buried food pellet on day 2 of the task (Noldus Ethovision). **i** Across all days except day 3, the ELS mice took similar time to find the pellet suggesting similar food detection capabilities between two groups of mice (*n* = 7−8 for both groups, *p* > 0.05 for all days except day 3 for which *p* = 0.03, Unpaired *t* test, two-tailed). **j** Scheme of novel object recognition task. **k** Tracks taken by control and ELS mice during testing. **l** The time explored was significantly more for novel (N) object for both groups of mice (*n*_C_ = 8, *n*_ELS_ = 7; *p*_C_ < 0.0001 for N vs. F and *p*_ELS_ < 0.0001 for N vs. F recognition task while *p* > 0.1 for the exploration time for N object, One-way ANOVA). **m** Object preference index was comparable between control and ELS mice (*p* = 0.66, Unpaired t-test, two-tailed). **n** Schema of carrying out c-Fos quantification following a perceptual learning task. d’ values between control and ELS mice differ, indicative of slower learning pace for distinguishing the binary mixture of Carvones (*n* = 6−7 per group, *p* < 0.0001, *F* = 25.4, Two-way ANOVA, LSD Fisher test). **o** Confocal images depicting c-Fos (red) and DAPI (green) expressi**o**n of control and ELS mice in the EPL (Scale bar = 20 μm). **p** Fewer c-Fos+ cells (per mm^3^) in the EPL were found in ELS mice after training on a complex odour discrimination task (*n* = 5−6 OBs from 3 mice each in both the groups; *p* = 0.013, Unpaired t-test, two-tailed). **q** Immunohistochemical expression of c-Fos and DAPI in the GCL (Scale bar = 20 μm). **r** Comparable c-Fos+ cells were observed in the GCL between control and ELS mice (*n* = 5−6 OBs from 3 mice each in both the groups; *p* = 0.22, Unpaired *t* test, two-tailed).

ELS groups of mice across different experiments were early weaned at P14 [29]. Throughout the nursing period from day of birth (P0), the litters with the dam were not disturbed, except a brief handling during weekly cage changing. Upon pre-weaning, the male pups were transferred to a new cage with new bedding and no nesting material (Fig. 1a). Food pellets were dissolved into a slurry and kept in a petri-dish. To avoid lethality due to hypothermia, the cage with pups were kept on the rodent heating pad, thrice in 24 h approx. for 1 h. This was done between P14 and P21.

### Olfactory go/no-go odour discrimination task under mouse freely moving condition

Mice’ behavioural training involved olfactory discrimination learning and memory tasks using our custom-built olfactometers, reported here is described elsewhere [27, 30].

#### Odours

Odours were bought from Sigma-Aldrich and mineral oil was purchased from Oswal pharmaceuticals, Pune, India. For odour discrimination learning and memory tasks, Acetophenone (AP), Octanal (ON), Cineole (CI), Eugenol (EU), Amyl acetate (AA), Ethyl butyrate (EB), Carvone-(+) (C+), and Carvone-(-) (C-) were used by diluting them to 1% (v/v) mineral oil. For quantifying the odour detectability of mice, AP/ON and AA/EB 60-40 odours were serially diluted in mineral oil starting from 10^-10^ % to 10^0^ %.

#### Paradigm

Odour discrimination learning task was an instrumental conditioning paradigm with one of the odours rewarded with water while other associated with no-reward. Mice were maintained on a 12 h water restriction schedule for maintaining motivation to perform the task. It was ensured that the weight of the mice never fell below 85% of their original weight while they were on the restriction schedule.

Each trial of the odour discrimination learning task began when the mouse poked into the sampling port guarded by an Infra-red beam. Odour is presented 500 ms after the beam breaks. Each trial can be presented after a fixed inter-trial interval of 5 s. For an odour which was rewarded (called S+ odour), mouse needed to lick on the lick tube continuously to get reward (3−4 µL per trial). The reaction or response window was kept for 2 s, divided into four bins of 500 ms each. For S+ odour, mouse needed to register a lick in at least any 3 out of 4 bins for getting the water reward. For a S- odour, mouse was allowed to lick in at most 2 out of 4 bins to carry out the trial correctly. Each block consisted of 20 trials, 10S+ and 10S-. Trials were sequenced in a pseudo-randomised fashion such that not more than 2 consecutive trials of the identical stimulus type were delivered. A total of 900 to 1200 trials were carried out per odour pair. On a single day, an optimally motivated mouse performed 80−120 trials in a single session of the task.

For probing olfactory memory, a resistance to memory extinction protocol was carried out as described elsewhere [28]. The protocol involved carrying out the last 100 trials of the training in a half-rewarded manner, i.e., only 50% of the S+ trials were rewarded randomly. One month later, the memory for this odour pair was assessed by interleaving the trials in the background of another learnt odour discrimination pair. The animal, if retained the memory, licked for the S+ memory odour and refrained to do so for S- memory odour. A total of 28 trials per mouse were interleaved in the last 140 trials of the background odour pair. Memory was quantified as the average accuracy achieved and represented as mean ± sem values.

#### Analysis

Learning curves were plotted by averaging the accuracy of 100 trials (50 S+ trials and 50 S- trials). Each datapoint corresponds to average accuracy achieved by the mice belonging to a group and thus, the learning curve is represented as mean ± sem values. Statistical significance was assessed by applying Two-way ANOVA followed by LSD Fisher test.

For calculating d-prime (d’), hit (correct S+) and false alarm (incorrect S-) probabilities were calculated over average of 100 trials per mouse. The z-score was calculated using the probabilities. d’ was quantified as z(hit)-z(false alarm) per 100 trials and plotted as mean ± sem values (Fig. 1d and Fig. S1). Statistical significance was assessed by applying Two-way ANOVA followed by LSD Fisher test.

### Open field test

Each trial of this test involved a mouse to explore the arena for 10 min. Before allowing the entry into the open field (OF) arena (60 cm x 45 cm), all cage-mates were habituated for 10 min before trial initiation in an arena similar to OF arena (dimensions were similar to home cage) connected via a small passage. Each mouse was allowed to enter the OF arena and exploratory behaviour was recorded and the different parameters were analysed using Noldus ethovision 8.5 (Fig. S5).

### Buried food pellet test

A modification of the common BFP test was carried out [31]. Mice were put on a food-restricted diet, i.e, consumption of food once in 24 h, which began 3 days prior to the start and continued during the 6-days of the BFP experiment. On each day of the 5-day testing, mouse carried one trial of finding the pellet buried 1.5−2 cm below the bedding (~3 cm thick layer). The pellet location was changed each day. The test was carried out in an arena of 60 cm x 45 cm dimensions and the bedding surrounding of the pellet was replenished after each trial. On day 6 of the experiment, a surface food-pellet test was carried out as a control for normal vision and locomotion of mice. The behaviour was recorded, analysed using Noldus ethovision 8.5 and the latency to find the BFP was statistically compared between the control and ELS groups for each day of the experiment (Fig. 1g–i).

### Novel object recognition test

This test comprised of two phases, a training or familiarisation phase and a testing phase separated by 1 h of delay [32]. During the training phase of 12 min, mouse was put into an arena and presented with two oppositely placed identical objects. In the test phase of 6 min, a third copy of the identical object used during the training phase (familiar object) and a novel object were placed in the same location as before. The positions of the two objects were counterbalanced between mice. The exploratory behaviour of mice was recorded and the time spent exploring the objects were analysed in Noldus ethovision 8.5 software. The preference index was determined by measuring the ratio of time exploring novel over the familiar object during the test phase (Fig. 1j–m).

### Immunohistochemistry and confocal imaging

Immunohistochemical experiments for analysis of c-Fos expression in the EPL and GCL of the OB was carried out after the mice were trained on an odour discrimination learning protocol for a binary 60%-40% mixture of carvone-(+) and carvone-(-) in an olfactometer under freely moving conditions (Fig. 1n–r). Quickly after training was finished, 3 mice from control and ELS groups each, were trans-cardially perfused with PBS and 4% PFA prepared in PBS. Brains were dissected out and stored in 4% PFA solution overnight at 4 °C. They were then transferred to the 30% sucrose solution for cryopreservation and kept for 24−48 h at 4 °C. Cryotome sectioning in a coronal manner, was carried out at 50 μm thickness and sections were collected in TBS in a 24 well plate. Three washes of 5-minutes with fresh TBS were performed. For IHC experiment, bulbar sections were selected at an interval of 300 µm from anterior to posterior direction. A blocking solution (7.5% NGS, 2.5% BSA and 1% Triton-X in TBS) was added for 4 h after which sections were immersed in a primary antibody blocking solution (Rabbit anti-cfos antibody, 2250 S, Cell signalling technologies) at a dilution of 1:500. They were incubated for 14 h at 4 °C in this solution. After giving 3 washes of 15-minutes using 0.5%-TBST, sections were incubated with the secondary antibody (Goat Anti-Rabbit Alexa flour 488, Jacksons Immunoresearch, 712-544-150, 1:1000 in a mix of 2% NGS, 1% BSA in TBS) for 2 h at room temperature. Once 3 washes with TBS were given after secondary antibody incubation, DAPI staining (1:500 diluted in 1% NGS solution of TBS) was carried out for 5 min. Stained sections were mounted on the glass slides using Vectashield (Vector Labs, H-1000) mounting medium and stored at 4 °C before imaging them. c-Fos signal was imaged in the EPL and GCL of the OB using Leica SP8 confocal microscope. To determine the number of c-Fos-positive cells in these two layers of OBs, Fiji ImageJ based and manual quantifications were done. The number was normalised to obtain cells per mm^3^ for each OB and statistically compared between the control and ELS mice using two-tailed, unpaired student *t* test. Confocal imaging of the SOM-GCaMP+ neurons of OB were carried out by injecting Dulbecco PBS solution during the perfusion process followed by vibratome sectioning the brain in Dulbecco PBS as followed elsewhere [33]. Chicken anti-NeuN antibody (ABN91, Merck, 1:1000) was used to co-stain the OB slices. The secondary antibody used was anti-Chicken AF647 (703-605-155, Jackson’s Immunoresearch, 1:1000). This allowed for enriching the GFP expression in the SOM neurons and thereby visualising it under confocal microscope.

### Morphological quantification of SOM interneurons

**Fig. 2:**
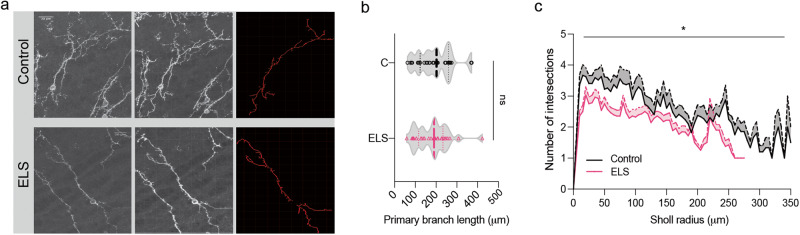
Morphological changes in the SOM inter-neuronal population of ELS mice. **a** Confocal images (left most) and the corresponding reconstructions (middle and in red) of the SOM-interneurons of the OB of young control and ELS mice (scale bar = 20 µm). **b** Quantification of the mean primary branch length of the SOM-interneurons suggests its similarity in the control and ELS mice (*n* = 29−31 SOM interneurons from 3 mice each in both groups; *p* = 0.43, Unpaired *t* test, two-tailed). **c** Sholl analysis indicates branching pattern aberrations in the SOM-INs of ELS mice (*n* = 27−28 SOM interneurons from 3 mice each in both the groups; *p* < 0.0001, Two-way ANOVA).

To quantify the primary branch length and the dendritic branching of the SOM interneurons of the OB, 3 young-aged control and ELS mice (SOM-transgenic) were utilised. Confocal imaging was carried out to image a total of 60 EYFP/GFP labelled SOM interneurons. For morphological quantification, 3D neuronal reconstruction was done using Imaris Software (Bitplane). This allowed measurement of primary branch length and the branching pattern was quantified using Sholl analysis on the re-constructed neurons [34]. In short, images were thresholded and soma was considered as the starting point for the Sholl analysis. Each sholl radius was increased by ~5 µm (Fig. 2).

### Slice electrophysiology

**Fig. 3:**
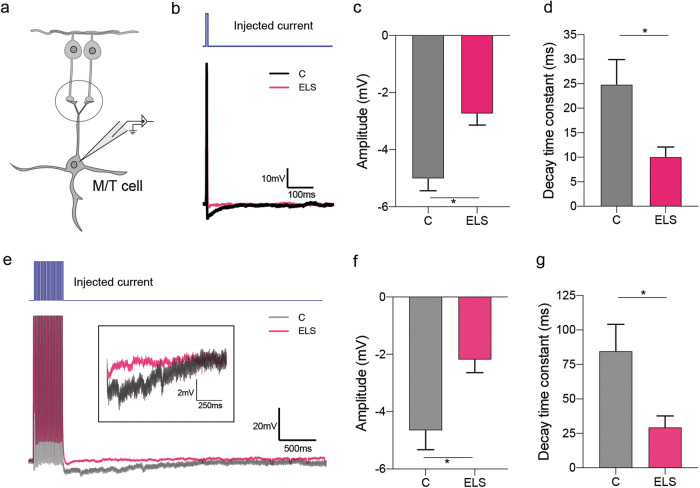
Strength of M/T cell inhibition is altered in ELS mice. **a** Whole-cell current clamp recording from the projection neuron (M/T cell) of the acute slices of olfactory bulb. **b** Representative electrophysiological traces from control (black) vs. ELS mice (pink) upon single step current injection. **c** Significantly reduced mean amplitude of the Inhibitory postsynaptic potential (IPSP) was observed in the ELS mice (mean A_C_ = −5.02 mV, *n*_C_ = 9 cells, *N*_C_ = 8 mice; mean A_ELS_ = -2.74 mV, *n*_ELS_ = 9 cells, *N*_ELS_ = 7 mice; *p* = 0.001, Unpaired *t*-test, two-tailed). **d** The IPSP decay time constant was lower in ELS mice (mean *T*_C_ = 24.89 ms, mean *T*_ELS_ = 10.14 ms; *p* = 0.014, Unpaired *t* test, two-tailed). **e** Representative electrophysiological traces from control (black) vs. ELS mice (pink) upon 20-pulse current injection. **f**, **g** Significantly reduced mean amplitude (mean A_C_ = −4.64 mV, *n*_C_ = 6 cells, *N*_C_ = 5 mice; mean *A*_ELS_ = −2.18 mV, *n*_ELS_ = 6 cells, *N*_ELS_ = 5 mice; *p* = 0.01, Unpaired t-test, two-tailed) and decay time constant (mean *T*_C_ = 84.52 ms, mean *T*_ELS_ = 29.26 ms; *p* = 0.02, Unpaired *t* test, two-tailed) in ELS mice.

**Fig. 4:**
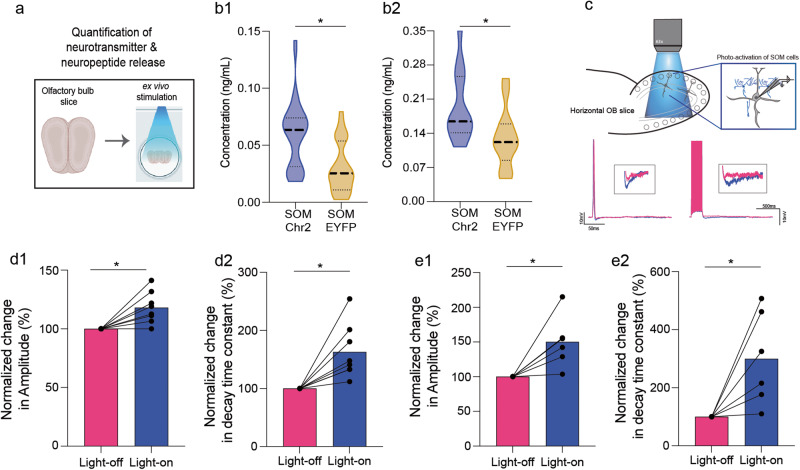
Enhancement in synaptic inhibition on M/T cells upon optogenetic activation of SOM-INs in ELS mice. **a** Ex vivo quantification of the neurotransmitter GABA and neuropeptide SOM upon optogenetic activation was achieved by photo-stimulating 150 μm OB oxygenated slices immersed in ACSF solution. After photo-stimulation was done, ACSF was collected and subjected to ELISA based quantification. **b1** Increased release of SOM neuropeptide was confirmed upon photo-stimulating the slices of SOM-ChR2 mice (*p* < 0.0001, Unpaired *t* test, two-tailed). **b2** GABA titre increased in the slices of SOM-ChR2 mice when the SOM interneurons were optically activated (*p* = 0.021, Unpaired t-test, two-tailed). **c** Illustration depicting preparation for electrophysiological recordings from M/T cells while the SOM-INs, expressing ChR2 are photo-activated in the bulbar slices from ELS mice. Below are the traces from ELS mice under light-off (pink) and light-on (blue) conditions upon single and multiple AP evoking protocols. **d1**, **d2** A significant increase is observed in the amplitude and decay time constant of IPSPs that are recorded upon photo-activating the SOM-INs in a single-AP protocol (*n* = 8 cells, *N* = 7 mice; p_Amplitude_ = 0.0068, p_Decay-time_ = 0.0067; Paired *t* test, two-tailed). **e1**, **e2** A significant increase is observed in the amplitude and decay time constant of IPSPs that are recorded upon photo-activating the SOM-INs in a 20-AP protocol (*n* = 6 cells, *N* = 6 mice; p_Amplitude_ = 0.021, p_Decay-time_ = 0.028; Paired *t* test, two-tailed).

Olfactory bulb was dissected from control and ELS mice of P30-45 age and immersed in an ice-cold, oxygenated slicing solution containing (in mM) NaCl 85, KCl 2.5, NaHCO3 25, NaH2PO4 1.25, Sucrose 75, MgCl2 3.3, CaCl2 0.5, glucose 25, ascorbate 0.4, pyruvate 2, myo-inositol 3, a sucrose-based Ringer solution before getting sliced. Horizontal slices (300 µm thick) were sectioned using a vibratome (Leica, VTS 1000) in an ice-cold oxygenated slicing solution. Upon sectioning, slices were transferred to an incubating bath maintained at 37 °C for 30 min for equilibration to room temperature. Finally, a slice was transferred to the recording chamber containing an extracellular artificial cerebrospinal fluid [(in mM) NaCl 125, KCl 2.5, NaHCO3 25, NaH2PO4 1.25, MgCl2 1, CaCl2 2, glucose 25]. Both the bath and recording solution was continuously bubbled with carbogen gas (95% oxygen and 5% carbon dioxide). Experiments were performed between 23 °C−26 °C. Electrophysiological recordings were done as described before [30, 35]. Whole cell, current-clamp recordings were achieved using multiclamp 700 B patch clamp amplifier and digidata 1440 A and pipettes with resistance of 4−6 MΩ filled with the solution containing (in mM) K methane sulphonate 130, HEPES 10, KCl 7, EGTA 0.05, Na2 ± ATP 2, Mg2 ± ATP 2, GTP 0.5, biocytin 0.4% (pH adjusted to 7.2 using KOH). Action potentials were evoked by short square current injections (generally 3 ms) with an inter-pulse interval of 8 ms (Fig. 3). To modulate the activity of SOM-INs, M/T cells were patched and the SOM-INs were stimulated by photo-activation by applying 40 Hz pulses of blue light (479 nm, DC4104 LED driver, Thor labs) while evoking the APs. In approx. 40% light stimulation trials of 20-AP generation, a total light stimulation of 500 ms was applied, and similar readouts were obtained (Fig. 4c–e). Analysis of the amplitude and the decay time constant of inhibitory postsynaptic potential (IPSP) was carried out by custom Python scripts. To calculate the decay time constant, bi-exponential function for curve fitting of the IPSP was used.

### In vivo calcium imaging

**Fig. 5:**
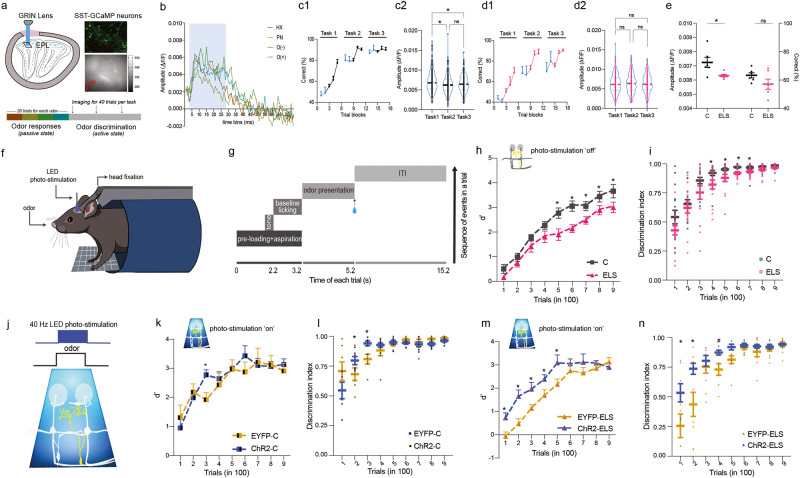
Optogenetic activation of bulbar SOM-INs rescues olfactory learning impairment in ELS mice. **a** Miniscope driven calcium imaging via a GRIN lens lowered into the external plexiform layer of the olfactory bulb of the SST-GCaMP6f transgenic mouse. Expression of SST-GcaMP6f in EPL under a confocal microscope (top right, green) and the signal recorded by the Miniscope (bottom right). Micro-endoscopic recording of the GCaMP fluorescence was carried out during odour detection (under anaesthetised conditions) and odour discrimination learning (awake state). The arrows indicate performing imaging for the first 40 trials in a single task for a total of three tasks. **b** Representative graph depicting the change in calcium fluorescence during the time of odour delivery (blue window). **c1**, **c2** Refinement in the activity of SOM-Ins of the OB occurs as the training progressed, on a simple Acetophenone vs. Octanal olfactory discrimination task (*n* = 6 SOM-GCaMP mice; *p* = 0.0068, Kruskal-Wallis test, recorded trials data points consist of 40 trials, non-recorded trials are plotted as 80-80-100 trials). **d1**, **d2** Similar activity profiles of SOM-INs as the odour discrimination tasks progressed indicates their lower recruitment during learning in ELS mice (*n* = 6 SOM-GCaMP mice; *p* = 0.865, Kruskal-Wallis test). **e** ELS mice depict a reduced involvement of SOM-INs during the initial phase of learning period (p_Amplitude_ = 0.03, p_Accuracy_ = 0.15; Unpaired *t* test, two-tailed). **f** Head-restrained set-up for the optogenetic modulation of the circuitry while animals were performing an odour discrimination task using an automated olfactometer. **g** Paradigm of olfactory discrimination task in a head-restrained set-up (ITI: inter trial interval, blue drop: water reward for S+ trials). **h** Odour discrimination task without any photo-stimulation. ELS mice (i.e., both ChR2-ELS and EYFP-ELS mice), under no photo-stimulation, exhibited slower olfactory learning pace as compared to control mice (combining ChR2-C and EYFP-C groups) (*n* = 15 mice per group, *p* < 0.0001, *F* = 37.42, Two-way ANOVA, LSD Fisher test). **i** Discrimination index was faster for control mice (*p* = 0.002, *F* = 10.76, Two-way ANOVA, LSD Fisher test). **j** Frequency of blue light (479 nm) stimulation at 40 Hz was achieved by using a T-cube LED driver connected to a LED implanted on the cranial window over the bulbar area. Stimulation was done for 2 s, during the odour presentation window. **k** Upon optogenetic activation of bulbar SOM interneurons in control mice, a minute increase in the learning pace of ChR2-C mice was observed for a complex binary mixture of Carvones (*n* = 7 mice per group, *p* = 0.37, *F* = 0.79, Two-way ANOVA, LSD Fisher test, *p* < 0.05 for a data point marked with asterisk). **l** The differences in AUC plot represents a marginal increase during the initial phase of discrimination learning in ChR2-C mice (*p* = 0.8, *F* = 0.03, Two-way ANOVA, LSD Fisher test, *p* < 0.05 for data points marked with asterisk). **m** Upon photo-activating the SOM interneurons of the OB of ChR2-ELS mice, faster learning was achieved as compared to EYFP-ELS mice (*n* = 5−8 mice per group, *p* < 0.0001, *F* = 25.27, Two-way ANOVA, LSD Fisher test). **n** A significant enhancement in the discrimination index was observed for ChR2-ELS mice during the early learning phase (*p* < 0.0001, *F* = 17.22, Two-way ANOVA, LSD Fisher test, # = *p* < 0.1).

Imaging cannula (GRIN lens assembly, Doric Lens Inc.) was inserted into the craniotomy hole drilled in the centre of the right OB of SOM-GCaMP mouse. Dura was removed from the area where the lens was placed. The protrusion adjustment ring was screwed on the lens assembly such that upon lowering the lens into the tissue, it reaches to the depth of EPL of OB where the neuronal cell bodies lie. Dental cement mixed with cyanoacrylate gum was applied to stabilise the assembly. Each mouse was singly housed after the surgery completion. A snap-in fluorescence microscope body (465 nm from the LED source, OSFM Model L, Doric Lens Inc.) was mounted on the imaging cannula during the recordings (Fig. 5a). Calcium imaging experiments began ~25 days after the surgery. The frames were acquired at 10 Hz for 10 s for each trial and the field of view was 350 µm × 350 µm (further binned at 2 × 2 times) and then onset of imaging was synchronised with the odour delivery by external TTL signal generated by the olfactometer. Odour responses were recorded after anaesthetising the mice by injecting ketamine/xylazine intraperitoneally (Fig. 5b). For odour discrimination learning of AP vs. ON, 40 trials per task were recorded (Fig. 5c–e). Using Open CV python library, relative change in fluorescence, ΔF/F was measured for each trial. This was done by subtracting the baseline fluorescence (initial 1.2 s) from all time bins.

### Measurement of GABA and Somatostatin release

To confirm the release of neurotransmitter GABA and neuropeptide SOM upon optogenetic activation, we carried ex vivo OB slice preparation from SST-ChR2 and SST-EYFP mice using the methodology used elsewhere [36]. In brief, mouse was anesthetized and decapitated, and the brain was dissected out within few minutes of sacrificing the animal. Dissected brain was immersed in high-sucrose modified artificial cerebrospinal fluid solution (194 mM sucrose, 20 mM sodium chloride, 4.4 M potassium chloride, 2 mM calcium chloride, 1 mM magnesium chloride, 10 mM glucose, 1.2 mM sodium phosphate monobasic, 26 mM sodium hydrogen carbonate in distilled water, pH 7.4). Coronal sections of OB were taken at 150 μm thickness, in an ice-cold, continually oxygenated high-sucrose solution using a vibratome (Leica VT 1000 S). Slices were transferred to a holding chamber in a normal ACSF (124 mM sodium chloride, 4.4 M potassium chloride, 2 mM calcium chloride, 1 mM magnesium sulphate, 10 mM glucose, 1.2 mM sodium phosphate monobasic, 26 mM sodium hydrogen carbonate in distilled water, pH 7.4) maintained at 31 °C and constantly bubbled with carbogen. Slices were shielded from light until photo-stimulation protocol was employed.

Multi-LED assembly was custom-built for photo-stimulation of slices kept in a 12-well plate (Fig. 4a). Two modules of 6 LEDs each connected in series were fabricated. Voltage drop across each LED was measured to calculate the total voltage required to power the whole assembly of the modules connected in parallel. A MOSFET connected to an Arduino Nanoboard was used steady maintenance of voltage and operating the assembly at 40 Hz frequencies of light stimulation. Light was switched on for 2 s followed by shutting off for 13.2 s duration, which is the inter-trial interval we kept for optogenetic behaviour experiments. The modules were assembled on the lid of a 12-well plate and the LEDs were lowered to achieve maximum proximity to the OB slices.

At least 4 pairs of OB slices per mouse were utilised for photo-stimulation. Two pairs of OB slices were transferred to each well of a 12 well plate containing 500 μL of oxygenated ACSF solution. Photo-stimulation protocol was done for 20 min post which, 3 × 50 μL of the solution per well was collected in ice-cold tubes. Once all samples were ready for peptide quantification, ELISA assay was immediately employed. 96-well Somatostatin-14 ELISA kit (S-1179, BMA, Biomedicals, Switzerland) and GABA ELISA kit (MOFI01269, AssayGenie) were utilised and the assay was performed. Briefly, 50 μL of freshly prepared standards or samples were incubated with 25 μL of anti-serum in buffer for 1 h. Biotin tracer (50 μL) was added to this and left at room temperature for 2 h. After washing with the buffer, 100 μL streptavidin-HRP was added and incubated for 1 h. After 5 more thorough washes, 100 μL TMB solution and the reaction was terminated by adding 100 μL of 2 N HCl. Absorbance was measured at 450 nm using Perkin Elmer Multimode Plate reader. Quantification was done from the standard curves, reported in ng/mL range for SOM & GABA (Fig. 4b1, b2).

### Mouse surgery for optogenetic behavioural experiments

Headpost implantations were carried out before implanting LEDs over the OB of mice belonging to control and ELS groups (P50-P60 old). SOM-EYFP, SOM-ChR2 and SOM-Arch mice from both the groups were utilised to carry out optogenetic behavioural experiments. Mice were anesthetized using a mix of Ketamine and Xylazine (50 mg/kg and 10 mg/kg, respectively) and mounted on the stereotactic instrument. After removing the skin overlying the skull, periosteum was gently peeled off by using scalpel blade and fine forceps. After cleaning the skull surface using freshly prepared ACSF solution, etching agent (Ivoclar Vivadent EcoEtch) was applied for a few seconds and cleaned thoroughly afterwards. After air-drying, dental primer (Ivoclar Vivadent Te-Econom bond) was applied and polymerised by showing UV light flashes for 30 s. As a final step, dental cement (Ivoclar Vivadent Tetric-N-Ceram) was applied on the exposed area and UV polymerised. A headpost made out of stainless steel was fused using the dental cement and once it was placed in the desired angle, UV polymerisation was done to adhere it to the skull. The surrounding area was covered by the dental acrylic cement (DPI RR Cold cure, acrylic repair material). After 2 days of recovery from the surgery, mice were put on the head-restrained olfactometers and the stability of the headposts was checked.

Next, the headpost-implanted mice were subjected to cranial window creation and LED implantation surgery after ensuring that their weights are maintained stably. Skin overlaying the OB was removed. The surgical cut was extended to 1 mm anterior of OB. The periosteum was gently peeled and the exposed surface was washed with cortex buffer. A biopsy punch of 2.5 mm was used to create a hole over the central area of the two OBs and a hand-held drill was employed to gradually deepen the grove. Once the thinned bone groove was made, the central bone island was lifted without damaging the dura so as to finally form the cranial window. Cortex buffer was applied continuously while creating the window and gelfoam (Abgel, SGK labs, Mumbai) was applied to coagulate bleeding. A drop of dexamethasone was put on the dura to prevent inflammation. A glass coverslip of 3 mm was placed on the cranial window. Once the surrounding skull was air-dried, dental acrylic cement was put to seal the coverslip in place. A hot-melt gun glue drop was placed over the coverslip once the surgery was done. After the visibility through the coverslip was ensured 4−6 days post-surgery, LED (blue LED: NFSB036BT and orange LED: NJSA172, Nichia corporation) connected with a header-socket arrangement (ED11100-ND, ED8250-ND, Digikey) was implanted over it. It was fixed in its place by using the dental acrylic cement. Behavioural experiments ensued after 3−5 days of recovery from the surgical procedures (Fig. 5f).

### Olfactory go/no-go odour discrimination task under head-restrained condition

Mice’ head-restrained behaviour involved odour discrimination learning and memory tasks under ‘light-off’ and ‘light-on’, i.e., optogenetic photo-stimulation off and on conditions, respectively (Fig. 5f, g). This was done as described elsewhere [28, 37, 38].

#### Odours

Odours were bought from Sigma-Aldrich and mineral oil was purchased from Oswal pharmaceuticals, Pune, India. For odour discrimination learning and memory tasks, Acetophenone (AP), Octanal (ON), Carvone-(+) (C+), Carvone-(-) (C-), Amyl acetate (AA) and Ethyl butyrate (EB) were used by diluting them to 1% (v/v) mineral oil.

#### Paradigm

Task habituation or pre-training was carried out in a similar way as described elsewhere [28, 38]. Briefly, after the water-restricted mice were comfortable while being restrained, they were trained to sequentially learn the aspects of licking on the lick tube to get reward, waiting for the reward by minimising the licking during pre-loading period and gradually increasing the time of licking for getting the reward. An odour detection task of 40 trials ensued at the end of pre-training. Once the cranial window & LED implantations were done successfully, LED power was decided on a single animal basis by training them on odour detection task. This facilitated determining the least LED power (ranging 2−13 mW/mm^2^) for SOM-ChR2 and SOM-Arch mice that could result in similar licking probability as under photo-stimulation ‘off’ condition. Once it was determined, same power was used during the discrimination learning task.

Odour discrimination trial consisted of odour presentation of 2 s after the pre-loading time of 3.2 s (Fig. 5g). To ensure that the mouse did not associate the LED visual cue during the odour presentation with the reward, a background LED was suspended few cm above the animal, which switched on at the starting of pre-loading time. Licking criteria for S+ and S- odours were same as followed elsewhere [28]. The tone signal was kept on only during odour detection phase of pre-training. Water restriction schedule was followed on a 12-h basis per day.

#### Analysis

The d’ measurements were done the similar way as noted above. For calculating Area under the curve (AUC) for lick probability curves for S+ and S- every 100 trials for a total of 900 trials, custom-written Python scripts were run. To calculate discrimination index from the AUCs of lick curves, following formula was used: AUC = (AUC_S+_ - AUC_S-_)/ AUC_S+_.

### Statistics

Behavioural data analysis was done using IgorPro, custom Python scripts, video analyses using Noldus EthoVision and statistical analyses was performed in GraphPad Prism. In different experimental methods used in this study consistently provided statistical significance between group comparisons, with a minimum number of 6 experimental animals. The animal numbers are reported for all data sets. All data is represented as mean±sem values and were within 2*SD. Normality of the data was checked using the Shapiro-Wilk test. Unpaired two-tailed *t* test, Ordinary one-way ANOVA, Two-way ANOVA with associated *posthoc* tests, Gehan-Breslow-Wilcoxon test and Kruskal-Wallis test were done to assess the statistical significance. For all statistical analyses, *p* < 0.05 was considered statistically significant.

## Results

### Early life stress exacerbates olfactory learning and memory of male mice

Early weaning disrupts mother-infant interactions during the late-lactation phase, which causes deprivation of olfactory and other sensory cues provided by the mother to the pups [39]. This can exert adverse effects on sensory brain regions that controls decision-making behaviours [40]. Here, we investigated the behavioural and neurophysiological changes that may arise upon ELA via early weaning. Owing to the neuroanatomical closeness and top-down neuromodulation that the OB receives, we specifically focused on behaviours pertaining to sense of smell. We began behavioural phenotyping during their young adulthood (at P60) by investigating their perceptual learning and memory skills using an odour discrimination paradigm (Fig. 1a). Experimental and control mice were trained to discriminate simple monomolecular as well as their complex binary mixtures (complex odour pairs achieved by mixing them in 60%−40% ratio) on a go/no-go conditioning paradigm [27, 30, 38, 41]. The accuracy of animals’ odour discrimination performance was quantified based on the sampling and licking readouts toward the rewarded (S+) and non-rewarded odour (S-) stimuli (Fig. 1b and see methods). The d-prime (d’) was measured for each animal as the parameter for quantifying it’s performance on a perceptual olfactory discrimination learning task and usually the d’ value increased as learning progressed (Fig. 1c). Upon carrying out 900 trials of discriminating Acetophenone (AP) vs. Octanal (ON) odours as well as its binary mixture, slower learning pace was observed for ELS mice as compared to the control group (Fig. 1d). We probed their memory of odour-reward association one month after the discrimination training for another odour pair, Amyl acetate (AA) vs. Ethyl butyrate (EB) and it’s binary mixture (Please see Fig. S1 for the complete training sequence). Poor olfactory memory was observed in ELS mice (Fig. 1e). To substantiate their learning deficits, we combined the learning performances for different simple and complex stimuli. We found out a significant increase in the number of training blocks that ELS mice took to reach high accuracy (Fig. 1f).

To study if the early weaning caused any alterations in their olfactory detection abilities, we performed two tests. On carrying out BFP test across 5 days of burying the pellets at different locations in the arena, we did not observe any difference in the food detection abilities for ELS mice compared to normally weaned, control mice (Fig. 1g–i). Further, we trained another cohort of control and ELS mice on odour pairs (AP vs. ON and AA vs. EB binary mixture) at different dilutions, starting from 10^-8^ to 10^0^ % (v/v) [41]. Both groups started to discriminate AP vs. ON at 10^-4^ % and AA vs. EB binary mixture at 10^-6^ %, however, the pace of learning continued to be slower for ELS mice (Fig. S2a and b). This confirmed that the odour detectabilities for ELS mice was normal and can be postulated that learning as well as memory deficits we observed with ELS mice is not due to their compromised detectabilities. To investigate if animals are using any non-specific cues during the discrimination learning, we carried out Mineral oil vs. Mineral oil (diluent used for odour dilution) discrimination task, which resulted in chance level learning for both groups (Fig. S3). As ELA can lead to cognitive deficits, we tested their learning and memory on a novel object recognition task (Fig. 1j). Upon quantifying the novel object preference index 1 h after training with familiar objects, similar exploratory times were exhibited by ELS and control mice (Fig. 1l, m). This revealed that early weaning paradigm we used did not induce disabilities in discrimination learning pertaining to other sensory modalities (Fig. 1k–m). ELA induced cognitive deficits can persist throughout the entire lifetime. We carried out olfactory discrimination training in a subset of control and ELS mice during late adulthood. Slower learning pace was observed, with experimental mice compared to control ones, proving the long-lasting olfactory learning deficits induced by ELS (Fig. S4). However, their anxiety-like responses quantified by open field test (OFT) did not occur during the late adulthood (Fig. S5).

To unravel the neural underpinnings of the olfactory learning deficits in ELS mice, we first examined the neural activation pattern using c-Fos marker in the OB, the first relay centre in the rodent olfactory pathway. Mice were trained on a discrimination task of complex binary mixture of carvones and immediately sacrificed at the end of training (Fig. 1n). This allowed us to decipher learning- dependent c-Fos activation in the OB of ELS and control mice (Fig. 1o, q). While the number of learning-induced c-Fos+ cells were comparable in the GCL of control and ELS mice (Fig. 1r), they were significantly reduced in the EPL of the ELS mice, indicating the role of EPL interneurons in the observed olfactory learning impairments (Fig. 1p).

### Reduced synaptic inhibitory feedback in M/T cells and altered dendritic morphology in SOM-INs of OB of ELS mice

The EPL of the OB is populated by Somatostatin (SOM)-releasing GABAergic interneurons, which are shown to be vulnerable under various stress conditions, in higher brain areas [22]. They are also sparsely present in the GCL of the OB. However, their contribution in the synaptic inhibitory feedback to the projection neurons under ELS conditions and thereby refining the output from the OB remains elusive. Upon morphological characterisation of this subset of interneurons at P16-P18 age (Fig. 2a), we did not find any difference in the length of the primary dendritic branches, however, found an overall reduction in the branching pattern of the interneurons on carrying out Sholl analysis (Fig. 2b, c). This hint towards a possible alteration in the synaptic physiology of projection neurons as SOM-INs neurons make functional synapses with them [23, 24].

We further probed if ELS induces alterations that affect the functionality of projection neurons of OB, namely the Mitral/Tufted (M/T) cells. To this end, we quantified the inhibitory feedback on M/T cells by measuring the hyperpolarization of the membrane potential, which is shown to be resulting mostly from the recurrent inhibition [30, 42]. Whole-cell current clamp recordings were done from the M/T cells in the horizontal sections (300 μm) of the OB (Fig. 3a). Single/multiple current injections were done to evoke the APs in M/T cells and the hyperpolarization of the membrane was quantified (Fig. 3b, e). To quantify the extent of Inhibitory postsynaptic potentials (IPSPs) from the M/T neurons in the OB slices from control and ELS mice, IPSP amplitudes and the decay time constants were computed. A decrease in the mean IPSP amplitude and IPSP decay time constant of ELS M/T cells were observed in case of single (Fig. 3c, d) as well as multiple (Fig. 3f, g) APs generation. Thus, modulation elicited by the inhibitory network was significantly weaker in the OB circuit of ELS mice. This provides an explanation for the learning deficit we observed with ELS mice as the decreased inhibition can cause reduced refinement of olfactory information, which results in slower learning pace [37].

### Enhancement of synaptic inhibition by photo-activation of ChR2-expressing SOM-INs in ELS mice

To investigate if the morphological deficits in SOM-INs are translating to functional changes in the circuitry that may influence the reduced synaptic inhibitory feedback, we optogenetically modulated their activity in ELS mice while patching the M/T cells. Firstly, to ensure that light frequency and time utilised for ChR2 photo-activation was physiologically-relevant and resulted in the neurochemical release, we employed ex vivo method of photo-stimulation followed by ELISA-based measurement of SOM and GABA [36]. A custom-built multi-LED array allowed simultaneous activation of ChR2 in OB slices (Fig. 4a). The released peptide/neurotransmitter in the ACSF solution was subjected to ELISA based quantification (See methods). Photo-activation resulted in the enhanced release of GABA and SOM from the OB slices of SOM-ChR2 mice compared to control (SOM-EYFP) animals (Fig. 4b1, b2). Upon confirming the release of neurochemicals, we began opto-electrophysiological experiments. Similar approach of electrophysiological recordings (as in Fig. 3a) was employed. The M/T cells were patched and the SOM-INs were stimulated by photo-activation by applying 40 Hz pulses of blue light (479 nm, DC4104 LED driver, Thor labs) while evoking the APs. We elicited APs by applying brief current injections and recorded the hyperpolarizing responses in presence and absence of photo-stimulation for the same cells (Fig. 4c). We found a significant increase in the amplitude and the decay time constant of the light-evoked IPSPs (Fig. 4d1–e2). The increase in synaptic inhibition onto the M/T cells that occurs upon activating SOM-INs suggests that these interneurons are actively involved in modulating the output of the OB under condition of ELS. The light-evoked increased inhibition supports the involvement of bulbar SOM-INs in mediating the effect.

### Optogenetic activation of bulbar SOM-INs rescues learning deficits of ELS mice

As photo-activating SOM-INs brought about an increase in the synaptic inhibition of the M/T cells, we decided to track their activity during the behaviour to get a direct readout of the learning-dependent changes. To this end, we tested the changes in the Ca^2+^ dynamics from SOM-IN population. We integrated the miniScope-enabled calcium imaging with the olfactometer from SOM-GCaMP6f mice, under head-restrained conditions (see methods, Fig. 5a). Before we quantified the Ca^2+^ activity from behaving mice, we tried to observe it under anaesthesia, as these neurons are sparsely located in the EPL (Fig. 5b). We carried out a simple odour discrimination training, AP vs. ON, for three tasks, interleaving the recording trials in each task (Fig. 5a, c1). Imaging in control mice revealed an overall reduction in the amplitude of the normalised fluorescence changes as the learning progressed from task 1 to task 3 (Fig. 5c2). This indicated learning-dependent refinement in the activity of SOM-INs. On the other hand, similar amplitude levels over the course of training were observed in ELS mice (Fig. 5d2), suggestive of a potential reduction in the synaptic activity, which was reflected by their altered morphology. Further, reduced amplitudes in ELS mice during the initial learning phase indicates lowered recruitment of SOM-INs as compared to the control mice (Fig. 5e).

To investigate the regulatory role of bulbar SOM-INs in driving ELA dependent changes in the perceptual learning, we employed bidirectional optogenetic modulation of the neural circuitry in the control and ELS mice while the animals were performing the odour discrimination task. Having observed modulation of the inhibitory feedback on the projection neurons by SOM-INs, we first employed ChR2 mice for specifically activating these INs while they underwent behavioural training. We started with training different cohorts of mice: SOM-EYFP-Control (SOM-EYFP-C, control for light stimulation), SOM-ChR2-Control (SOM-ChR2-C, for comparing the photo-activation effects with ELS mice), SOM-EYFP-Early life stressed (SOM-EYFP-ELS, for comparing the photo-activation effects with ELS mice) and SOM-ChR2-ELS (experimental group), for a simple AP vs. ON discrimination task (photo-stimulation off) under head-restrained conditions (Fig. 5f, g). Upon comparing the C and ELS mice, we observed slower learning pace with ELS mice similar to what we observed under freely moving conditions. As their learning efficiency was computed based on their licking behaviour, we quantified another discrimination index (DI), the normalised difference in the AUC of lick probabilities for S+ and S- odours (see methods). ELS mice showed lowered DIs compared to control animals (Fig. 5h, i, refer S6a for the representative lick probability curves). Upon confirming the behaviour phenotype of ELS mice under photo-stimulation off conditions, we next carried out SOM-ChR2 activation at 40 Hz frequency for the duration of odour delivery (2 s) while the animals were actively performing the task of discriminating binary mixture of carvones (Fig. 5j). While only a marginal change in the learning and discrimination index of ChR2-C mice (Fig. 5k, l, refer S6b for the representative lick probability curves) was observed, the ChR2-ELS mice exhibited a faster proficiency to learn a complex odour discrimination task as measured by d’ and discrimination index parameters (Fig. 5m, n, refer S6c for the representative lick probability curves). The rescue of learning deficiency on photo-stimulating SOM-INs in ELS mice confirms better refinement of olfactory representations caused by enhanced inhibition.

### Optogenetic inhibition of SOM-INs leads to poor perceptual learning

**Fig. 6:**
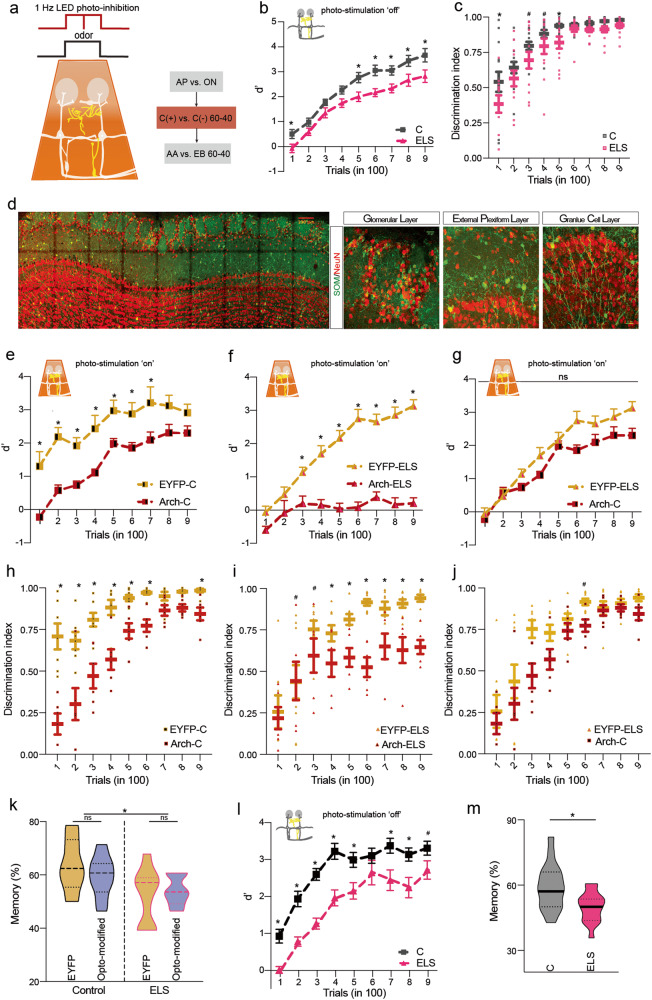
Optogenetic inhibition of SOM-INs in control mice phenocopies ELS-mediated learning impairment. **a** LED (590 nm) firing at 1 Hz frequency was utilised for performing photo-inhibition of the bulbar circuitry in SOM-Archaerhodopsin (Arch) mice while they were performing the complex binary mixture task of Carvones. This was preceded by AP vs. ON task and proceeded with AA vs. EB binary mixture task, carried out under photo-inhibition ‘off’ conditions. **b** ELS mice (belonging to EYFP-ELS and Arch-ELS groups) performed at a slower pace in an AP vs. ON discrimination task carried out in a head-restrained olfactometer when optogenetic inhibition was not applied (*n* = 13−15 mice per group, *p* < 0.0001, *F* = 50.50, Two-way ANOVA, LSD Fisher test). **c** Discrimination index (obtained from lick probability curves, see Fig. S5) for control and ELS mice (*n* = 13−15 mice per group, *p* < 0.0001, *F* = 20.38, Two-way ANOVA, LSD Fisher test). **d** Confocal images of SOM-GCaMP tagged neurons across different layers of the OB (tile scan scale bar = 100 µm and other images scale bar = 20 µm). **e** Photo-inhibition of bulbar circuitry controlled by the SOM neurons in Arch-Control mice (maroon) leads to impaired olfactory perceptual learning when compared to EYFP-Control group (yellow) (*n* = 6−7 mice per group, *p* < 0.0001, *F* = 68.06, Two-way ANOVA, LSD Fisher test). **f** Upon photo-inhibiting the circuitry, Arch-ELS mice displayed chance-level performance for a complex olfactory discrimination task (*n* = 6−8 mice per group, *p* < 0.0001, *F* = 296.7, Two-way ANOVA, LSD Fisher test). **g** Mimicking of ELA-induced learning deficit phenotype was observed in Arch-C mice when compared to EYFP-ELS mice (*n* = 6-8 mice per group, *p* < 0.0001, *F* = 20.18, Two-way ANOVA, LSD Fisher test). **h** Slower discrimination in Arch-C mice (*n* = 6−7 mice per group, *p* < 0.0001, *F* = 127, Two-way ANOVA, LSD Fisher test). **i** Arch-ELS mice exhibited poor discrimination of the S+ and S- odours upon photo-inhibiting SOM interneurons (*n* = 6-9 mice per group, *p* < 0.0001, *F* = 39.9, Two-way ANOVA, LSD Fisher test). **j** The discrimination capabilities were comparable between Arch-C and EYFP-ELS mice (*n* = 6-9 mice per group, *p* = 0.005, *F* = 11.45, Two-way ANOVA, LSD Fisher test, *p* > 0.05 for all data points). **k** Olfactory memory formation for carvones binary mix remains unaffected when checked 1 month after the training (*p* = 0.01 for EYFP-C vs. EYFP-ELS; *p* > 0.5 for comparison within controls or within ELS sub-groups, Ordinary one-way ANOVA). **l**, **m** Learning and memory for the next odour pair in sequence, AA vs. EB binary mix, remains altered for ELS mice (combining all 3 optogenetic groups), under photo-stimulation off conditions (*n* = 18−19 mice per group, *p* < 0.0001, *F* = 85.26, Two-way ANOVA, LSD Fisher test for learning and *p* = 0.0053, Unpaired *t* test, two-tailed; for memory).

Having achieved the rescue of learning deficiency by photo-stimulating the SOM-INs, we sought out to study the opposite modulation by stimulating inhibitory rhodopsin in them. Optogenetic inhibition of these INs was achieved by utilising SOM-Arch mice expressing light-activated archaerhodopsin protein in the SOM-INs. The continuous light activation was carried by using 590 nm LED placed over the OB while the animal was performing the complex odour discrimination task (Fig. 6a). We first investigated the learning of SOM-EYFP-Control (SOM-EYFP-C), SOM-Arch-C, SOM-EYFP-ELS and SOM-Arch-ELS for a simple odour discrimination task. EYFP mice of both groups were common between the two sets of experiments of optogenetic activation and inhibition. The learning deficiency quantified by d’ and DI prevailed in the ELS mice (Fig. 6b, c, refer S7a for the representative lick probability curves).

When the SOM-INs across OB layers (Fig. 6d) were photo-inhibited throughout the course of olfactory discrimination training in Arch-C mice, it caused the learning deficiency, quantified by d’ and DI, in control mice on a complex task, which mimicked the ELS phenotype (Fig. 6e–j, refer S7b, c for the representative lick probability curves). The photo-inhibition of SOM-INs in ELS mice caused severe phenotype, where a complete loss of learning was observed (Fig. 6f, i). Although the learning was bi-directionally modulated by optogenetic activation and inhibition, the memory formation was unaffected by these modifications (Fig. 6k). Further, the memory deficiency was confirmed with ELS mice using another odour pair, binary mixtures of AA vs. EB (Fig. 6l, m). Together these data demonstrate that SOM-INs of the OB play a role in ELA-induced olfactory discrimination learning deficits.

## Discussion

While limited evidences exist on the malfunctioning of sensory perception induced by ELA, our study dissects out the negative effects on the olfactory perceptual learning and memory caused by ELA and the underlying neural circuit mechanism. ELA predisposes individuals to psychological and cognitive disturbances [6, 43]. Understanding the underlying neural mechanisms becomes a challenging task since sensory as well as higher cortical areas along with limbic system are involved in generating behavioural disorders [1, 44]. Much of the ELA-related literature in the field focused on the changes mediated by hypothalamus, hippocampus and amygdala [2, 45–47]. However, our interest in probing learning and memory deficits pertaining to olfaction stemmed from the knowledge of prevailing bi-directional connectivity between olfactory sensory areas and the stress-sensitive brain regions [19]. Moreover, the presence of neurons that release stress-vulnerable neuropeptides, glucocorticoid receptors, and susceptibility of adult-born OB neurons to stressors allowed us to hypothesise OB-dependent behavioural changes in ELS mice [14, 23, 48, 49]. Here, we focused on one of these factors, i.e., the role of SOM releasing GABAergic interneurons in mediating ELS-dependent changes. Our results demonstrate the relevance of these neurons present in the OB in modulating the adverse consequences of ELA.

Upon observing that the ELA-induced deficits were specific for olfactory learning and memory, and not arising due to impaired odour detection, we observed a reduction in the c-Fos activity in the EPL of the olfactory bulb of trained mice (Fig. 1). We focused our attention on the functionality of SOM-INs and found alteration in their morphological features which indicated their impaired action under ELS (Fig. 2). Indeed, on carrying out whole cell recordings from the M/T cells that control the output from OB, which is refined by GABAergic signalling, proved altered synaptic inhibition caused by ELA. The decreased M/T cells inhibition correlated with the compromised odour discrimination accuracy of early weaned mice (Fig. 3). Involvement of SOM-INs in controlling as well as enhancing the synaptic inhibition on the M/T cells of stressed mice was confirmed using opto-electrophysiology (Fig. 4). SOM-INs' role in modulating the odour information was further confirmed by the refinement of Ca^2+^ activity during the discrimination learning phase of control mice (Fig. 5). Finally, by optogenetically modifying the activity of the SOM-INs and thereby controlling the inhibition on projection neurons of the OB, we noticed the rescue of learning deficits upon photoactivating the INs under ELA. Conversely, photo-inhibiting SOM-INs decelerated the learning pace in control mice, which mimicked the ELA deficits, and completely abolished discrimination learning in the ELS mice (Figs. 5 and 6). Our study thus provides evidences for the affective-state dependent perturbation of OB circuitry. However, to alleviate the compromised memory under ELA conditions, we may have to probe beyond OB circuits or target specific bulbar synapses that can undergo experience-dependent modifications [50, 51].

Different ELA models can affect the maternal-pup interactions to varying extent, which are dependent on the duration and severity of the treatment. In the maternal separation (MS) paradigms where pups are returned to the dam after brief intervals can result in the enhanced nursing which may buffer the evoked stress. In contrast, our paradigm of early weaning during the late lactation period causes severe phenotypes as the perturbations happen during the stress hyper-responsive period [39]. This is carried out during the period that can interfere with the functioning of cortico-limbic circuits, which may ultimately lead to the altered inputs to OB under ELA [44]. The involvement of basolateral amygdalar regions in mediating behavioural deficits was revealed by a modified GNG paradigm. After the training, animals were tested for the rewarded stimuli provided in an anxiety-evoking environment, where ELS mice showed slower latency towards the reward [47]. However, in our paradigm, the training and testing were carried out in an anxiety-free environment, as we wanted to study the perceptual learning. We have successfully used this paradigm elsewhere to study the effect of altered olfactory representations in the OB on modifying perceptual learning [30, 37, 38].

Even in human studies, OB volume was reduced in the patients who suffered childhood maltreatment and also under MDD conditions [12, 13]. In such neuropsychiatric and degenerative disorders, SOM-INs across multiple brain regions are affected [22, 52]. However, their role in modulating olfactory functions under ELA condition remains elusive. Within the OB, SOM-INs are located in key positions, i.e GL, EPL, and GCL, to modulate the synaptic network (Fig. 5d). As they are present in different locations, there can be inhibitory synaptic connectivity established at different compartments of projection neurons’ dendrites (example: apical vs. lateral). Our results from slice physiology experiments showing lowered synaptic inhibition in ELS mice and its enhancement upon photo-activating SOM-INs which further confirms their role in refining the output from OB and thereby controlling the learning efficiency of animals. Although the task-dependent refinement quantified by Ca^2+^ imaging was observed in control mice, the overall changes in the amplitudes were low. As the SOM-IN soma lie sparsely in the EPL with extensive branching pattern, it is hard to detect fluorescence directly from the cell-bodies with small field of view (FOV)-miniscopes. With the advanced optical imaging techniques; single-cell Ca^2+^ imaging by utilising newer open-source large-FOV miniscopes or large scale miniaturised two-photon microscopes are needed to probe the Ca^2+^ dynamics of SOM-INs at single cell resolution. In addition, the dissection of layer-specific role of SOM-INs of OB by employing three-photon microscopy are tangible goals in the near future [53–55]. In view of different somatostatin receptor expression patterns in the different layers of OB, future studies are needed to dissect out the modulatory peptidergic versus GABAergic role of SOM-INs in the olfactory functions [20, 56]. Many clinical reports over last two decades indicate various olfactory problems in human subjects caused by anxiety and depressive states [12, 40, 57]. Indeed, these states can be induced by ELA too. Therefore, quantifying olfactory fitness of the ELA-affected population using precise methods may help in identifying and characterising sensory and cognitive deficits at early stages and help in designing better treatment strategies [58–62].

OB circuits are critically involved in modulating socio-sexual behaviour of humans [63]. The ELA effect on these behaviours is less explored. In mice, the spatio-temporal specific deletion of oxytocin receptors from anterior olfactory nucleus resulted in the top-down modulation of OB projection neurons and problems in recognising conspecifics [64]. However, the extent of ELA effects on olfactory subsystems that are involved in pheromonal information processing remains elusive. Experiments using sensitive behavioural assays quantifying pheromonal-based perceptual learning would provide answers to many of these related questions [65, 66]. Taken together, our findings provide evidences for the negative effects of ELA on the pre-cortical brain area, the OB, and the perceptual learning deficits caused by it. By using a multi-pronged approach comprising optogenetics, electrophysiology, and in vivo population imaging, we revealed the responsible neural circuit and succeeded to rescue the learning dysfunctions, which may facilitate designing therapeutic approaches involving a stress-vulnerable interneuron population.

### Supplementary information

Supplementary Information
