# Supplementary material for: Perceptual learning deficits mediated by somatostatin releasing inhibitory interneurons of olfactory bulb in an early life stress mouse model
Source: Mol Psychiatry. 2023 Sep 19;28(11):4693–706. doi: 10.1038/s41380-023-02244-3 (PMC10914616; doi:10.1038/s41380-023-02244-3)
Supplement: Supplementary file 1 — Supplementary Information [file 41380_2023_2244_MOESM1_ESM.pdf]

Supplementary Information

Supplementary figures

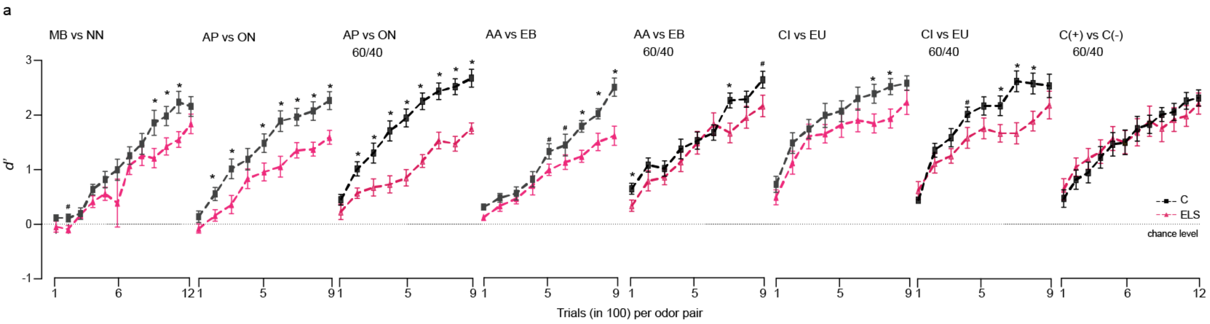

Fig. S1 Sequence of odour pairs utilized for olfactory learning assessment of ELS mice. a d' plots across different odour pairs done in the sequence represented in the figure (N=17-21 mice per group, $p < 0.05$ for all data points marked with *, Two-way ANOVA, LSD Fisher test). Methyl Benzoate (MB), Nonanol (NN), Acetophenone (AP), Octanal (ON), Amyl acetate (AA), Ethyl butyrate (EB), Cineole (CI), Eugenol (EU), Carvone-(+) (C+), and Carvone-(-) (C-).

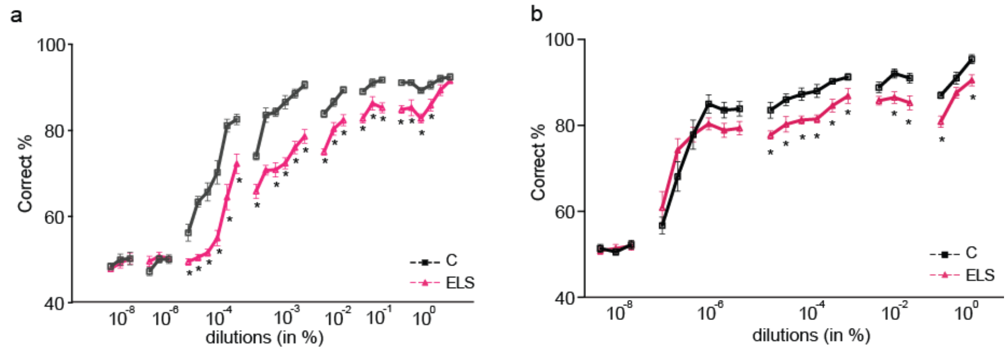

Fig. S2 Thresholds of discriminating odours is normal in ELS mice. a Odour discrimination learning for simple odour pair AP vs. ON starting from a non-perceivable dilution 10^{-8} % (v/v) to 1%. Both groups start to perceive and learn the task from 10^{-4} % dilution, however, the learning still remains compromised in ELS mice ($n_c = 7$, $n_{ELS} = 8$; $p < 0.001$, $F = 438.8$, Two-way ANOVA, LSD Fisher test, $p < 0.05$ for data points with asterisk). **b** A binary mixture task of AA vs. EB was also carried out. Both ELS and control mice started to distinguish from 10^{-6}% suggesting normal odour detection ability in ELS mice. However, the learning pace remained slower for ELS mice from 10-4% to 1% dilution range ($p < 0.001$, $F = 38.23$, Two-way ANOVA, LSD Fisher test, $p < 0.05$ for data points with asterisk).

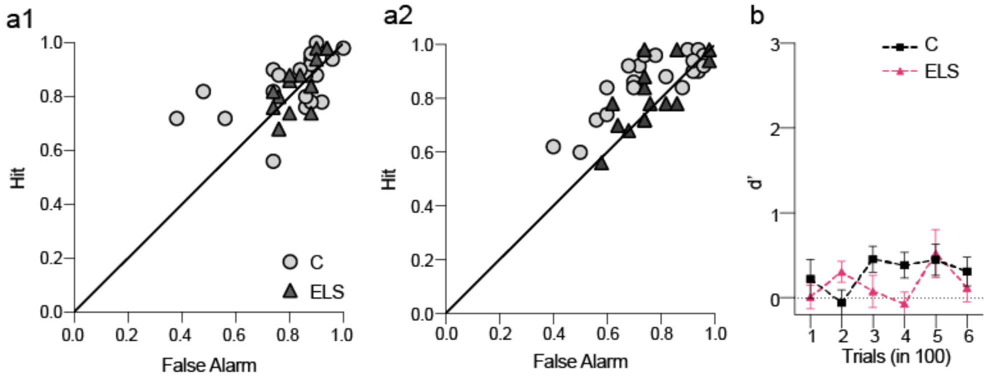

Fig. S3 Olfactory based learning is not influenced by any other sensory cues in our paradigm. a1,a2 Hit vs. False alarm probabilities are comparable throughout two tasks training for a Mineral oil vs. Mineral oil task with d' centring around 0, for both groups. **b** Comparable d' between control and ELS mice ($n_c = 7$, $n_{ELS} = 5$; $p = 0.2$, $F = 1.62$, Two-way ANOVA).

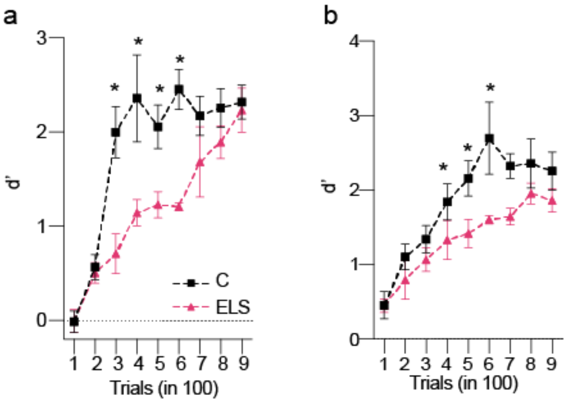

Fig. S4 Odour discrimination learning in the late-adulthood of mice. a d' plots for AP vs. ON for control and ELS mice ($n_c = 6$, $n_{ELS} = 5$; $p < 0.0001$, $F = 33.27$, Two-way ANOVA, LSD Fisher test). **b** d' for carvones binary mixture discrimination task ($n_c = 6$, $n_{ELS} = 5$; $p < 0.0001$, $F = 19.21$, Two-way ANOVA, LSD Fisher test).

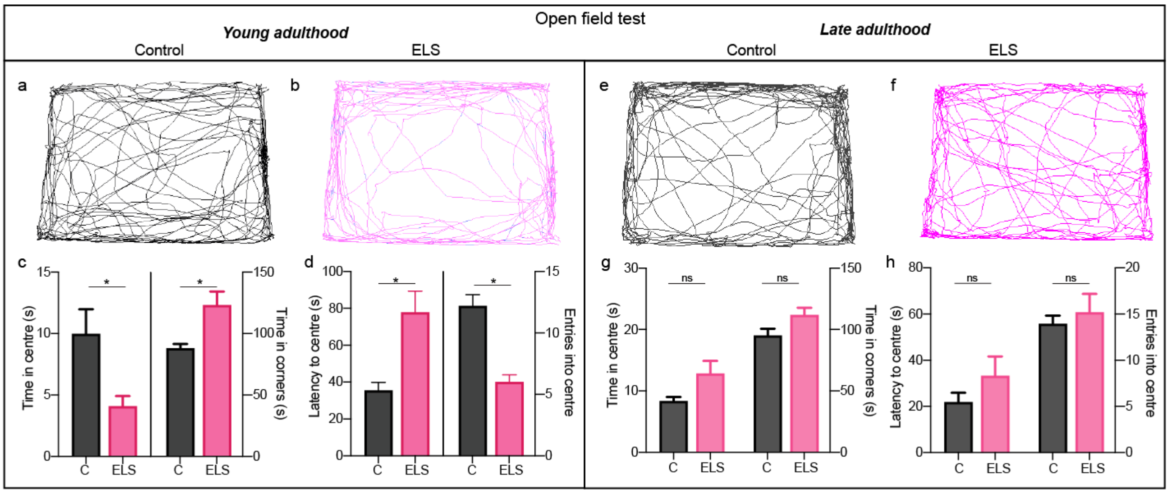

Fig. S5 Anxiety-like responses induced by ELS did not persist in the late-adulthood of mice. a,b Representative track of a control (black) and ELS (pink) mouse in an open field test in their young age. **c** ELS mice spent significantly lesser time in the centre ($n_c = 10$, $n_{ELS} = 8$; $p = 0.024$, Unpaired t-test, two-tailed) and more time in the corners of the arena as compared to the control mice ($p = 0.004$, Unpaired t-test, two-tailed). **c** ELS mice took longer time to make first visit ($p = 0.0018$, Unpaired t-test, two-tailed) in the central area and entered significantly fewer number of times as compared to the control mice ($p < 0.0001$, Unpaired t-test, two-tailed). **e, f** Representative track of a control (black) and ELS (pink) mouse in an open field test in their late adulthood. **g** Both groups of mice spent comparable amount of time in central zone and corners of the arena ($n_c = 10$, $n_{ELS} = 8$; $p > 0.05$, Unpaired t-test with Welch's correction, two-tailed). **h** The latency and total entries made into the centre were not statistically different between two groups ($p > 0.2$, Unpaired t-test with Welch's correction, two-tailed).

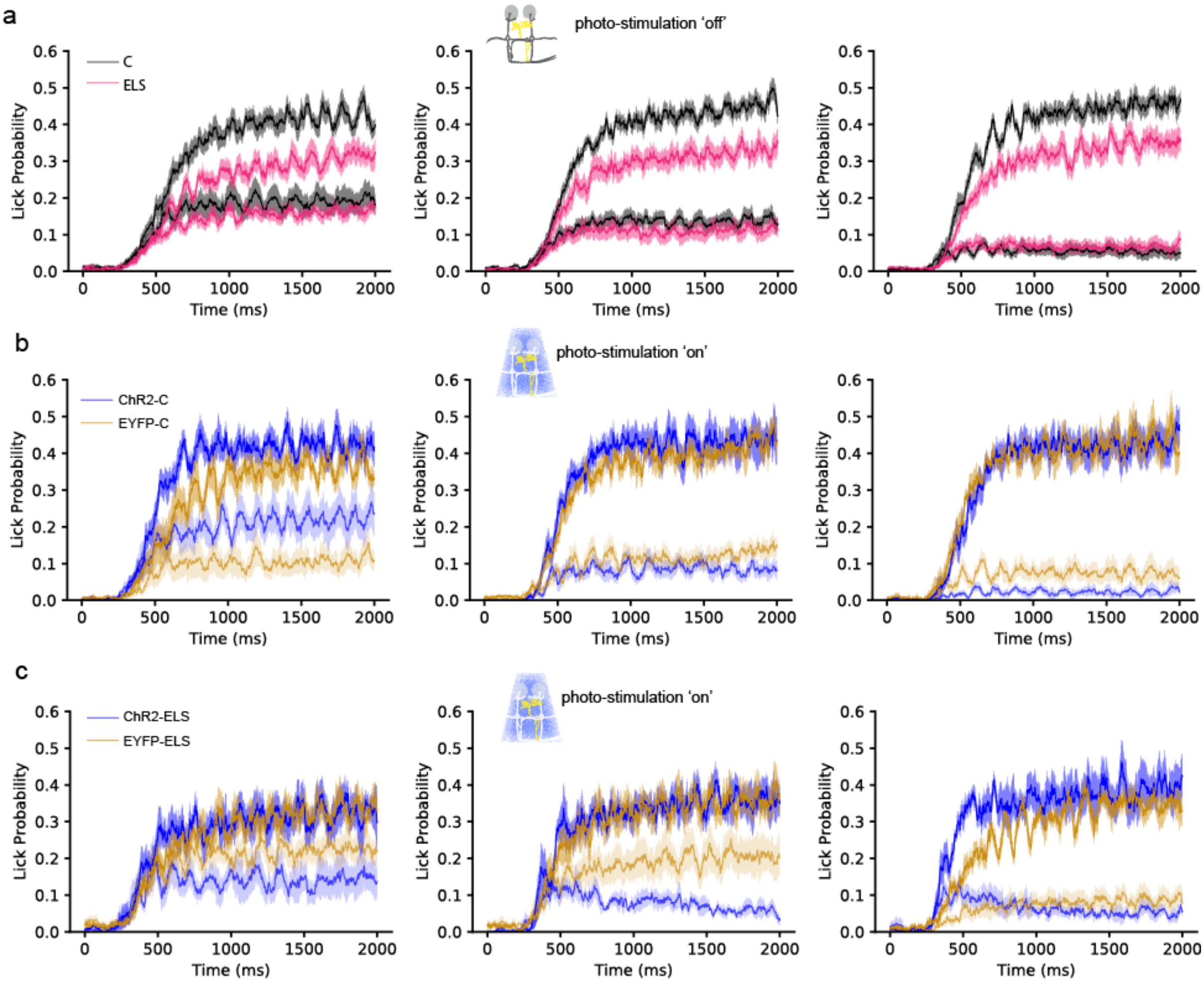

Fig. S6 Lick Probability curves indicate changes in the discrimination capabilities upon photo-activating SOM-INs. a Licking for S+ and S- odours change, i.e, increases for S+ and decreases for S- as the task progresses. This brings about the divergence and thereby, the changes in the AUC for each curve, introduced as the Discrimination index in Figure 5i. We observed faster divergence achieved by control mice (black curves) as compared to ELS mice (magenta curves) for AP vs. ON discrimination task, carried out under photo-stimulation off conditions. **b** Lick probability improves slightly in control mice upon carrying out photo-activation of SOM-INs (blue curves) for Carvones binary mixture task. Quantification of these are shown in Figure 5l. **c** Lick probability curves diverge faster in ChR2-ELS mice (blue curves) as the learning progresses, under photo-stimulation 'on' conditions. Quantification of these are shown in Figure 5n.

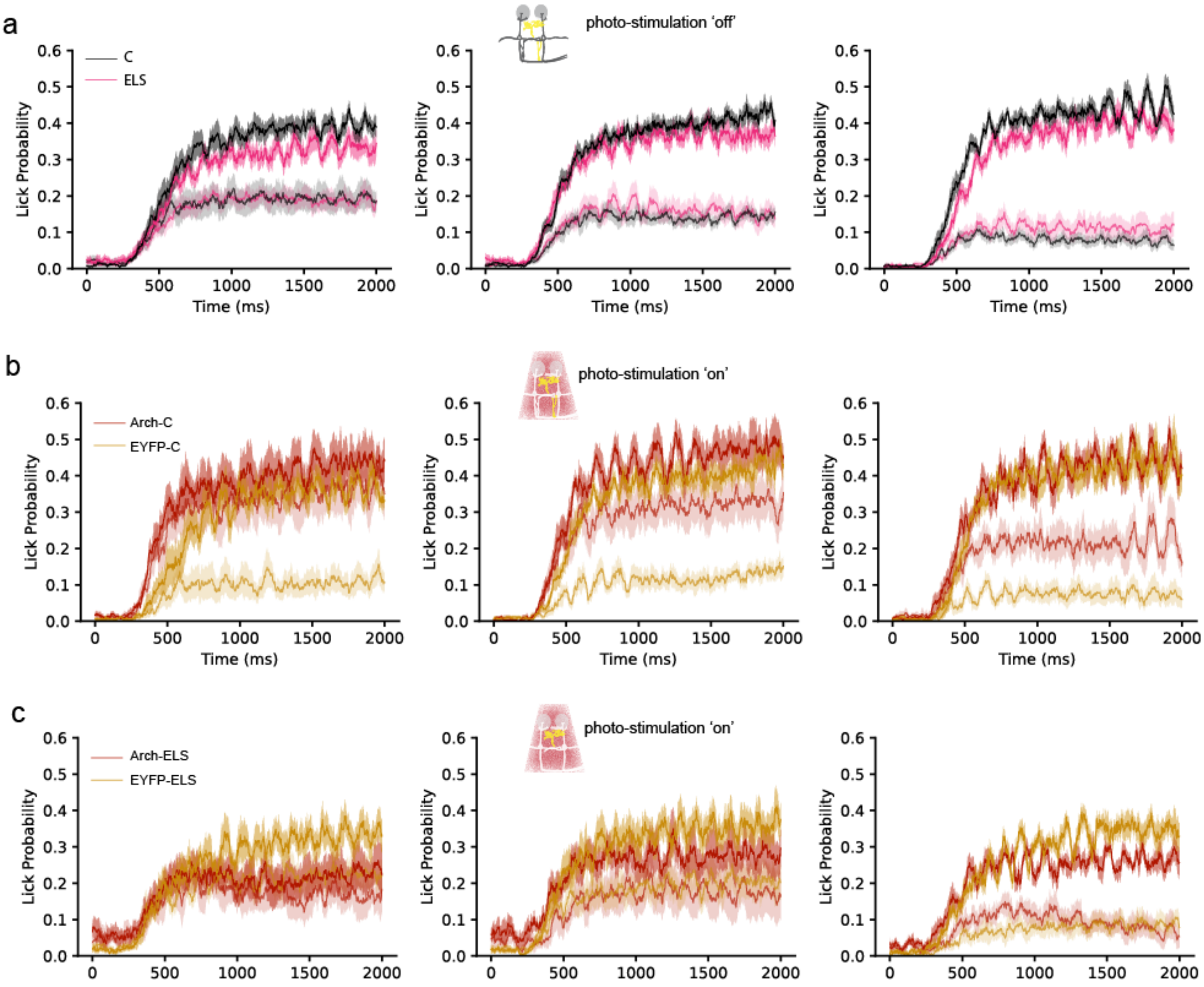

Fig. S7 Lick Probability curves reflect slower discrimination upon photo-inhibiting the SOM-INs. a Lick probability curves for control (black) and ELS mice (magenta) for AP vs. ON carried out under photo-stimulation off conditions. Animals belonged to SOM-EYFP and SOM-Arch groups. Quantification of these are shown in Figure 6c. **b** Discrimination happens at a slower rate when the SOM-INs are photo-inhibited as observed in the lick probability curves for Arch-C mice (maroon). Quantification of these are shown in Figure 6h. **c** Similar observations were made for Arch-ELS mice as well (maroon) as compared to the EYFP-ELS mice (Brown) Quantification of these are shown in Figure 6i.
